# Effectiveness of telerehabilitation programme following surgery in shoulder impingement syndrome (SIS): study protocol for a randomized controlled non-inferiority trial

**DOI:** 10.1186/s13063-017-1822-x

**Published:** 2017-02-23

**Authors:** Jose-Manuel Pastora-Bernal, Rocío Martín-Valero, Francisco Javier Barón-López, Oscar García-Gómez

**Affiliations:** 1 Health Science, Degree of Physiotherapy, University of Málaga, Málaga, Spain; 2 Department of Physiotherapy, Faculty of Nursery and Physiotherapy, PhD Lecture University of Cádiz, Cádiz, Spain; 3 Department of Physiotherapy, Faculty of Health Sciences, PhD University of Málaga, Málaga, Spain; 4 Rehabilitation Department, Hospital Costa del Sol, Málaga, Spain; 5 Department of Physiotherapy, Faculty of Health Sciences C/Arquitecto Francisco Peñalosa Ampliación del C. Teatinos, 29071 Málaga, Spain

**Keywords:** Telerehabilitation, SIS (shoulder impingement syndrome) physiotherapy, Surgery procedure, Telemedicine

## Abstract

**Background:**

Shoulder pain is common in society, with high prevalence in the general population. Shoulder impingement syndrome (SIS) is the most frequent cause. Patients suffer pain, muscle weakness and loss of movement in the affected joint. Initial treatment is predominantly conservative. The surgical option has high success rates and is often used when conservative strategy fails. Traditional physiotherapy and post-operative exercises are needed for the recovery of joint range, muscle strength, stability and functionality. Telerehabilitation programmes have shown positive results in some orthopaedic conditions after surgery. Customized telerehabilitation intervention programmes should be developed to recover shoulder function after SIS surgery. The objective of this study is to evaluate the feasibility and effectiveness of a telerehabilitation intervention compared with usual care in patients after subacromial decompression surgery.

**Methods:**

We will compare an intervention group receiving videoconferences and a telerehabilitation programme to a control group receiving traditional physiotherapy intervention in a single-blind, randomized controlled non-inferiority trial study design.

**Discussion:**

Through this study, we will further develop our preliminary data set and practical experience with the telerehabilitation programmes to evaluate their effectiveness and compare this with traditional intervention. We will also explore patient satisfaction and cost-effectiveness. Patient enrolment is ongoing.

**Trial registration:**

ClinicalTrials.gov, NCT02909920. 14 September 2016.

**Electronic supplementary material:**

The online version of this article (doi:10.1186/s13063-017-1822-x) contains supplementary material, which is available to authorized users.

## Background

Shoulder pain is common in society, with 7–27% of the adult population experiencing shoulder pain at any one time, and 7–67% of people experiencing shoulder pain in their lifetime [1]. Shoulder pain is highly prevalent within the general population, along with back and neck pain. Studies suggest that shoulder impingement syndrome (SIS) is the most common cause of shoulder pain [2]; it is estimated that SIS accounts for 44–60% of medical visits for shoulder pain [3, 4].

A prevalence of 78 cases per 1000 inhabitants has been reported for SIS, and review studies relate variations in prevalence between 70 and 200 per 1000 adults, leading to a significant consumption of healthcare resources and productive losses due to employee absenteeism [5]. Approximately 20% of expenditures for disablement by musculoskeletal disorder are consigned to subjects with shoulder disorders [6].

A socio-economic study with 6 months’ follow-up in patients with shoulder pain estimated an average cost of 326 euros per subject in healthcare, and the total cost was 2069 euros taking into account the average economic loss associated with absenteeism [6].

SIS has been defined as the compression and mechanical abrasion of the rotator cuff structures as they pass beneath the coracoacromial arch during elevation of the arm [7]. Repetitive activity at or above the shoulder during work or sports represents the main risk factor for SIS. As with many shoulder disorders, increasing age also predisposes one to SIS [8]. Patients with SIS suffer from pain, weakness and loss of movement of the affected shoulder. Causes of impingement include acromioclavicular joint arthritis, calcified coracoacromial ligament, structural abnormalities of the acromion and weakness of the rotator cuff muscles [9].

This musculoskeletal disorder affects the structures of the subacromial space, which are the tendons of the rotator cuff and the subacromial bursa. Subacromial impingement syndrome appears to result from a variety of factors. Evidences exist to support the presence of the following anatomical factors: inflammation of the tendons and bursa, degeneration of the tendons, weak or dysfunctional rotator cuff musculature, weak or dysfunctional scapular musculature, posterior glenohumeral capsule tightness, postural dysfunctions of the spinal column and scapula and bony or soft tissue abnormalities of the borders of the subacromial outlet. These entities may lead to or cause dysfunctional glenohumeral and scapulothoracic movement patterns. These various mechanisms, singularly or in combination, may cause subacromial impingement syndrome [10]. However, recent literature suggests that SIS is, in fact, the final result of many shoulder diseases and can be regarded as a descriptive term for a broad spectrum of symptoms rather than a single diagnosis [11].

Initial treatment of SIS is predominantly conservative, including rest, non-steroidal anti-inflammatory drugs, corticosteroid injections, physical therapy and various forms of exercise and manual therapy. When symptoms persist for periods longer than 3 months, it is common to refer the case for a re-evaluation by an orthopaedic surgeon [12].

Surgical and non-surgical strategies are used to treat SIS. An article on the effectiveness of post-surgical interventions for SIS has already been published [13], and high success rates have been noted as a result of surgical procedures [14, 15]. The most common surgical intervention for impingement is subacromial arthroscopic decompression (SAD). A Cochrane review comparing SAD with an open surgery approach concluded that neither procedure has been shown to be superior to the other [16].

Post-operative exercise therapy is also recommended, although its effectiveness is less documented. Early progressive exercises (range of motion and strengthening exercises) have been shown to result in greater improvements in range of motion at 3 and 12 months than later dynamic and strengthening exercises. Reductions in pain were similar for both regimens [17].

There is scarce evidence regarding the effectiveness of exercise programmes after decompression surgery for subacromial impingement syndrome. Also, there is currently no consensus about the most appropriate post-operative exercise strategy. With this argument, a recent multi-centre randomized controlled trial in Denmark has concluded that a standardized physiotherapy exercise intervention resulted in statistically significant and clinically relevant improvement in shoulder pain and function at 12 months when compared to usual care [18].

In addition to traditional physiotherapy, telerehabilitation programmes have demonstrated their effectiveness, validity and non-inferiority and presented significant advantages in neurological, cognitive and musculoskeletal diseases, providing an opportunity to define new social policies and intervention.

Telerehabilitation is a term used to describe the provision of rehabilitation services at a distance using telecommunications technology as the service delivery medium [19]. It has also been defined as the remote delivery of rehabilitative services, such as monitoring, training and long-term care, using telecommunications technology [20]. Therapists are using the technology in a variety of ways. Some of these include therapeutic interventions, remote monitoring of progress, education and training delivered to families, access to rehabilitation professionals, coordinating care with other professionals and providing networking for individuals with disabilities [21].

In the last 15 years, telerehabilitation within the larger realm of telehealth has been used to aid rural communities, with an emphasis on older adults, to improve and access healthcare services with the objective of decreasing cost and transportation issues [22]. As this technique continues to grow in popularity, the number of clients benefiting has increased, and telerehabilitation is now recognized as a bridge between the medical professional and the client [23].

Telerehabilitation for musculoskeletal disorders has been studied, and interesting conclusions have been published: ‘Adding an Internet-based protocol is more effective than education and exercise alone for persistent hip pain’ [24]; ‘Standard musculoskeletal assessment of lumbar pain is valid via telerehabilitation’ [25]; ‘Interactive virtual telerehabilitation program is at least as effective as conventional therapy after total knee arthroplasty’ [26]; ‘Noninferiority of in-home telerehabilitation is an effective alternative to face-to-face service of patients following a total knee arthroplasty’ [27]; ‘In-home teletreatment seems to be a promising way to dispense rehabilitation services for proximal humerus fractures’ [28]; and last, ‘statistically significant improvements in exercise self-efficacy, mobility, quality of life, and patient satisfaction after 30-day hip fracture telerehabilitation’ [29].

Therefore, studies with telerehabilitation interventions should continue and improve methodologically, addressing new diseases and becoming oriented to results that can be validated, standardized and integrated into healthcare policies.

## Methods

The aim of this study is to evaluate the feasibility and effectiveness of a customizable telerehabilitation intervention compared with usual care in patients after subacromial decompression surgery. Secondary objectives are to identify satisfaction and perception of patients regarding telerehabilitation intervention and to evaluate the cost-effectiveness of the intervention. Our hypothesis is that the clinical outcomes of the telerehabilitation intervention after shoulder surgery for SIS will be effective and not inferior to traditional therapy.

**Fig. 1:**
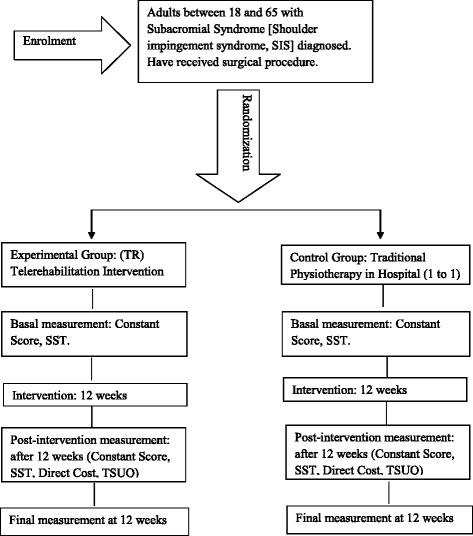
Study design

This is a single-blind, prospective, randomized clinical trial in the Rehabilitation service in Marbella’s Hospital Costa del Sol in Spain. This research project uses the guidelines on Standards for Quality Improvement Reporting Excellence (SQUIRE) [30]. A Standard Protocol Items Recommendations for Interventional Trials (SPIRIT) checklist is provided as Additional file 1, and the flow diagram for the study protocols is included as Fig. 1.

### Patients

The study includes adults between 18 and 65 years of age, diagnosed with subacromial syndrome (shoulder impingement syndrome, SIS) according to the 10th Revision of the International Classification of Diseases ICD-9 CM 726.10, 726.12, and 726.19 [31].

Subjects will have received a surgical procedure arthroscopically (subacromial decompression with partial acromioplasty, with or without coracoacromial release) or surgical codes related to SIS [14]. Furthermore, patients must live in Spain during the intervention phase, should have computer equipment with an Internet connection (including one of the following devices: personal computer, notebook, tablet or smartphone) that they can access frequently and they should have an existing email account. Exclusion criteria will be as follows: patients who have had surgery on the shoulder before the first contact with this research; those whose interventions are not based on surgical codes recommended for SIS; and subjects who do not have full cognitive abilities to allow the use of new technology tools. Table 1 lists the inclusion and exclusion criteria in detail.

**Table 1:** Inclusion and exclusion criteria

Inclusion criteria	Exclusion criteria
Adult between 18 and 65 years	Patients who received surgery in same shoulder before this research.
Subacromial syndrome diagnosis CIE-9 MC 726.10, 726.12, 726.19 issued by specialist in orthopaedic surgery or rehabilitation	Patients receiving surgical procedure non-based on the recommendations for subacromial syndrome
Receives surgical procedure (arthroscopy or open approach (subacromial decompression with partial acromioplasty, with or without coracoacromial release)) and prescription to start rehabilitation process.	Unfit cognitive ability to use technological tools
Lives in Spain during the investigation period	
Provides home computer with Internet technology (personal computer, laptop, tablet or smartphone)	
Skills and knowledge to access email	

The strategies for achieving adequate participant enrolment to reach target sample size include multidisciplinary collaboration of the Hospital Costa del Sol Team (Orthopaedic Surgeon, Head of Rehabilitation and physiotherapist’s team). The employees have been informed about the characteristics of the study via personal interviews and a project presentation.

### Randomization and single blinding

Before patient inclusion, a research secretary will generate the allocation sequence and randomly assign patients consecutively with opaque sealed numbered envelopes. We will use a computerized random number generator. The research secretary will be instructed not to reveal the allocation sequence to any of the research team. The nature of the intervention in both groups does not allow blinding of patients and physiotherapists. It is therefore a single-blind study, where the evaluator does not know the nature of the intervention.

### Intervention

Patients will be randomly assigned to (1) the Telerehabilitation (TR) group or (2) the Traditional Physiotherapy (PT) group. After randomization, patients in both groups will receive an initial evaluation. Data will be collected by a blinded evaluator and will be integrated into our research databases. The assessor and the patients will be instructed not to reveal the type of intervention they are using. The TR group will receive a customized exercises programme through a web application that allows the physiotherapist to generate videos, images and parameters of each exercise programme and send them via email.

The telerehabilitation programme describes the exercises to be performed, the number of repetitions (depending on the level of training) and criteria for progression. Patients will be initially supervised by a physiotherapist, who will conduct three individual videoconference training sessions (30 minutes each session) to ensure proper execution of exercises and encourage patient adherence.

Patients will be instructed to follow a 12-week (5 days/week) self-workout video exercise regimen following the guidelines of the telerehabilitation programme as well as a supporting document called the Telerehabilitation Patient Manual (this document is held by the corresponding author).

At baseline patients will receive advice on general care in physical activity, and issues concerning the intake of analgesics will be based on general practice and specialist advice. Patients will be warned not to practise any specific training for the shoulder during the intervention period (training not recommended by the physiotherapist). The physiotherapist will record any deviations from adherence and practice, noting any adverse impact on the exercises.

The PT group will receive assistance in the Costa del Sol Hospital’s physiotherapy department consisting of one-to-one physical therapy (manual therapy, home exercise programmes and other physiotherapy techniques) in a 12-week programme (5 days/week).

### Outcome measures

The initial assessment includes clinical interview and the following shoulder tests: the Constant–Murley Test (CM) (see Additional file 2) and the Simple Shoulder Test (SST) (see Additional file 3).

#### Primary outcome measures

At 4, 8 and 12 weeks, we will use the CM. The CM is an assessment tool universally used and accepted for shoulder function [32]. We will assess changes in the CM scores from baseline. The CM has a 100-point scoring system that is divided into four domains: pain (maximal 15 points), daily life activities (maximal 20 points), painless range of motion (maximal 40 points) and abduction strength evaluation (maximal 25 points) according to the methods described by Constant [33].

In addition we will use the SST Spanish validated version, an instrument that features 12 one-dimensional Yes/No answer questions [34]. The SST is a short questionnaire (2–3 minutes) — easy to understand and complete — and has validity and comparability with other subjective questionnaires [35]. The total score of 12 questions (2 related to pain, 7 on the force and 3 on the range of motion) [35], where 0 is the worst result and 100 is the best shoulder function measured, is calculated based on the number of positive responses multiplied by 100 [35]. The internal consistency of the test was measured by Cronbach’s alpha = 0.85 [36].

The impact of shoulder disorders can be evaluated from different perspectives. Traditionally, evaluation has been made at the local level, focusing on the functional aspects of the pathology and evaluating the range of motion, strength or pain [37]. Currently, there is an increasing trend towards the use of outcome measures that are subjectively reported by patients, assessing the perception of their own functional status [38]. These questionnaires are becoming widely used tools in the evaluation of treatment both in clinical practice and in medical research [34]. It is critical to employ valid and reliable research measures, and they must also be culturally and linguistically appropriate [39]. Current evidence supports the use of the SST questionnaire for longitudinal studies and clinical trials [34]. The SST is among the three subjective questionnaires that score higher reliability, responsiveness to change and interpretation [34]. In addition, the SST has been translated and cross-culturally adapted into Spanish with adequate psychometric properties [40].

#### Other pre-specified outcome measures

The acceptance and usability of telemedicine applications are prerequisites for identifying potential clinical benefits of this technology. Consequently, it is important to supplement this research with tools to examine the satisfaction and perception of patients [41]. We will use a Spanish adaptation of the Telemedicine Satisfaction and Usefulness Questionnaire (TSUQ) psychometric analysis, which supports the construct validity and internal consistency reliability and is available in English and Spanish [41] (see Additional file 4). This instrument has shown high reliability (Cronbach 0.8) and validity evidence regarding perceptions on telemedicine [42].

To evaluate the cost-effectiveness of telerehabilitation intervention, we will follow international guidelines for conducting cost analyses in randomized clinical trials [43]. This economic analysis is based on the perspective of the health sector, which means that only health intervention costs will be considered. Therefore, only the costs associated with the provision of health services in the traditional physiotherapy and the telerehabilitation group will be taken into account [44]. We will try to have similar baseline characteristics between the groups.

### Data collection and sources of data

Once patients are informed and randomly assigned to one group, we will be able to collect data for statistical analysis. This data collection will take place in the period from October 2016 to March 2017. An independent evaluator in the rehabilitation services will conduct initial assessment and the different measures at 4, 8 and 12 weeks. The data will be added to the database created for that purpose and administered by the principal investigator for statistical analysis.

### Statistical analysis

The trial results will be presented as a summary of the outcomes in each group, along with the estimated effect size and its precision. The statistical analysis will be performed in accordance with both an intent-to-treat analysis and a per-protocol analysis to have the maximum information about the treatment outcomes.

Frequency tables and histograms will present affiliation data for the participants. We will carry out a descriptive statistical analysis of the different variables, using frequency tables, bar charts and sector charts with the aim of having as much information as possible for exploration and analysis.

The study’s central hypothesis will be tested by comparing change in the CM score between the two groups for the intervention shoulder Appendix 1. To compare independent variables between the two groups, the Student’s *t* test will be used if validity criteria are met; otherwise, we will use the non-parametric approach Appendix 2. As other studies have shown [45], significant departures of normality are not expected. The difference in the evolution of the CM score between treatment groups from baseline to 4, 8 and 12 weeks follow-up will be tested using repeated measurements analysis of variance.

A non-inferiority study seeks to determine whether a new intervention is therapeutically equivalent, or not less equivalent, to an existing intervention reference [46]. In this study, the new intervention TR will be compared with the conventional standard of rehabilitation after SIS in Spain.

**Fig. 2:**
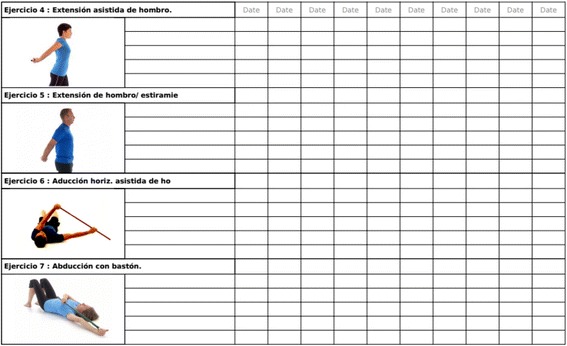
Example of customized telerehabilitation programme

Studies have presented results on the minimal clinically important differences for shoulder outcome measures (Fig. 2) [47, 48]. Recently, a study showed minimal detectable change ≥10 points in the CM score and a standard deviation (SD, 11.2) for patients with SIS [45]. Therefore, the non-inferiority criteria will be evaluated comparing the differences between groups with a non-inferiority margin of 10 points in the CM score, which is considered to be a clinically relevant change [49, 50].

Based on these data, we performed a calculation of the sample size. According to [45], an SD of 11.2 in the improvement of the CM would be expected. Anticipating a non-inferiority difference of 10 points on that test, a 90% power and the usual significance level of 5%, a sample size of 22 patients per group is needed. Anticipating a dropout rate of 15% (which will be studied by intention to treat), a total of 44 patients will be included (*n* = 44).

## Discussion

Telemedicine promises to improve quality, increase patient access and reduce costs in healthcare [51]. In addition, recent advances in telecommunication technologies have driven the possibility of rehabilitation processes through the Internet [52].

Studies have shown that telerehabilitation is effective in improving clinical outcomes in various diseases and have also found a strong positive effect for patients, especially after orthopaedic surgery, suggesting that increasing the intensity provided by the telerehabilitation is a promising option to offer to patients [52]. Shoulder pain is a common cause of sick leave and disability and therefore also results in a high consumption of healthcare resources and lost productivity [6]. This research should provide knowledge about the possibility of a new approach for the care of patients operated on for SIS, identifying telerehabilitation effectiveness and the patients’ level of satisfaction in order to finally analyse health resources and costs allocated to defining new policy intervention in this group of patients.

In contrast to other studies that require software implementations on specific devices, our intervention will generate few obstacles, as it is available on any device that allows Internet access, which patients usually have (desktop, laptop, tablet, smartphone). It thus allows access from different devices at any location. This contrasts with other studies that require highly complex technology platforms, software installation and multidirectional cameras for clinical control connecting the physical therapist and the patient [53]. Potential issues in the conduct of this study are the occurrence of selection bias and information. This study only includes individuals who have devices and access to the Internet. Future research should be undertaken to analyse whether individuals without access to the Internet have a different outcome. This research does not provide verification of socio-demographic and affiliation data provided by the participants, so we must assume reliability criteria for the information that they provide. Regarding the technological use, although videoconferences are widespread communication tools, they may present technical problems (disconnection, device failure) and technological difficulties. However, collaborative people are willing to offer support to patients by phone or email, without having to visit patients at home to install or verify any hardware.

Possible adverse events to be considered are a lack of improvement and positive developments in the range of movement, in terms of pain, muscle strength and function of the operated limb. Performing movements with excess load that cause tendon inflammation and muscle strain should also be considered as adverse events. Patients will be informed about the importance of warning the healthcare professional of any incident or misadventure in their recovery and their right to give up their participation at any time during the research.

### Trial status

Patient enrolment and recruitment are ongoing.

## Figures and Tables

**Fig. 3:**
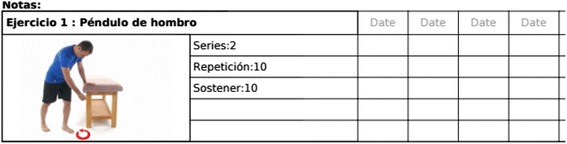
Notes and exercise description of telerehabilitation programme

**Fig. 4:**
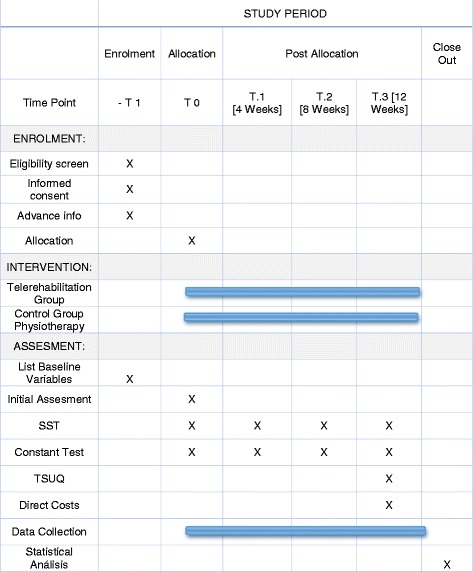
Schedule of enrolment, interventions and assessments

## Appendix I

### Previous Patient Information. English Version

WHY ME? Because you are older than 18 years and recently have undergone a surgical procedure on your shoulder. The Surgery and Rehabilitation team have found that you could benefit from the methods offered in this research project.

WHAT IS THE PROJECT ABOUT? Two interventions accepted and validated by the scientific community will be applied. Similar studies have shown that neither is inferior to the other in similar conditions, and we want to know if either of the two is better than the other, applying it rigorously as is explained in this form.

CAN I CHOOSE WHICH INTERVENTION TO TAKE PART IN? No. Allocation must be completely random, meaning you will be placed into one group or another by random selection, which is performed using a computer program. The research secretary will contact you to tell you which group you will belong to.

IF THE RANDOM SELECTION PLACES ME IN THE CONTROL GROUP, WHAT SHOULD I DO? You will be evaluated by an expert who will interview you and make an assessment (Fig. 3), using different scientifically validated tests and measures. This intervention lasts approximately 30 minutes. You will be re-evaluated at 4, 8 and 12 weeks, with the data on changes/progress recorded. Your physical therapist will take care of handling the recovery using the usual treatment.

IF RANDOM SELECTION PLACES ME IN THE EXPERIMENTAL GROUP, WHAT SHOULD I DO? You will be evaluated by an expert who will interview you and make an assessment, using different scientifically validated tests and measures (Fig. 4). This intervention lasts approximately 30 minutes. You will be re-evaluated at 4, 8 and 12 weeks, with the data on changes/progress recorded. The experimental group will receive a customized exercise programme performed through a web application that allows the physiotherapist to generate videos, images and the parameters of each exercise and then email it to the patients. The telerehabilitation programme describes the exercises to be performed and the number of repetitions, depending on the level of training and criteria for progression. You will be initially supervised by a physiotherapist via three videoconference sessions of 30 minutes each, to ensure proper execution of the exercises and to answer any possible doubts about the treatment to follow. Patients will be instructed to perform a self-workout following the exercise video using the telerehabilitation programme.

WHAT IF I DECIDE TO ALSO USE OTHER METHODS? Patients are warned to refrain from undergoing any specific training for the shoulder during the intervention period. Nothing will happen, but the research team must be informed if you undertake such action because of the influence it may have on the results.

AND AFTER 12 WEEKS OF INTERVENTION WHAT HAPPENS? In principle, the evidence shows us that 12 weeks may be sufficient for the recovery of your shoulder. Your physical therapist will recommend a maintenance regimen to follow after the end of the intervention period.

IF I DO NOT WANT TO FOLLOW IN THE STUDIO, WHAT HAPPENS? You can decide at any time to leave the project, simply by notifying the research group — no explanation will be necessary.

WHAT WARRANTIES DO I HAVE IF I CHOOSE TO PARTICIPATE? First, the project has been approved by the Ethics Committee of the Provincial Investigation of Malaga and the Ethics Committee of the Hospital Costa del Sol, so that intervention has to make maximum guarantees required for good practice and safety. In addition, there will be comprehensive monitoring by the principal investigator who is a professional physiotherapist with over 20 years of experience.

## Appendix II

### Patient Consent Form

Name and Surname:.....................................................................

1. I declare that I have read the Patient Information Sheet that accompanies this consent.
2. I was able to ask questions about the study. All questions were answered to my satisfaction.
3. I talked to the reporting healthcare professional:..........................................
4. I understand that my participation is voluntary and I am free to participate or not in the study.
5. I have been informed that all data obtained in this study will be kept confidential and will be treated according to the Organic Law of Protection of Personal Data 15/99.
6. I understand that I can withdraw from the study:
  - Whenever I want
  - Without having to explain
  - Without this impacting on my medical care

I freely give my agreement to participate in the project entitled

**Table:**

AGREE	DISAGREE
Patient Signature:	Healthcare Informant Signature
Name and Surname	Name and Surname
Date	Date

## Additional files

Additional file 1: SPIRIT checklist. (DOC 123 kb)
Additional file 2: Constant–Murley test, Spanish version. (DOCX 159 kb)
Additional file 3: Simple Shoulder Test (SST), Spanish version. (DOCX 124 kb)
Additional file 4: Telemedicine Satisfaction and Usefulness Questionnaire (TSUQ), Spanish version telerehabilitation adaption. (DOCX 35 kb)

## Abbreviations

CM: Constant–Murley test
PT: Physiotherapy group
SAD: Subacromial arthroscopic decompression
SIS: Shoulder impingement syndrome
SPIRIT: Standard Protocol Items Recommendations for Interventional Trials
SST: Simple shoulder test
TR: Telerehabilitation group
TSUQ: Telemedicine Satisfaction and Usefulness Questionnaire
