# Supplementary figures and images for: Effectiveness of telerehabilitation programme following surgery in shoulder impingement syndrome (SIS): study protocol for a randomized controlled non-inferiority trial
Source: Trials. 2017 Feb 23;18:82. doi: 10.1186/s13063-017-1822-x (PMC5324280; doi:10.1186/s13063-017-1822-x)

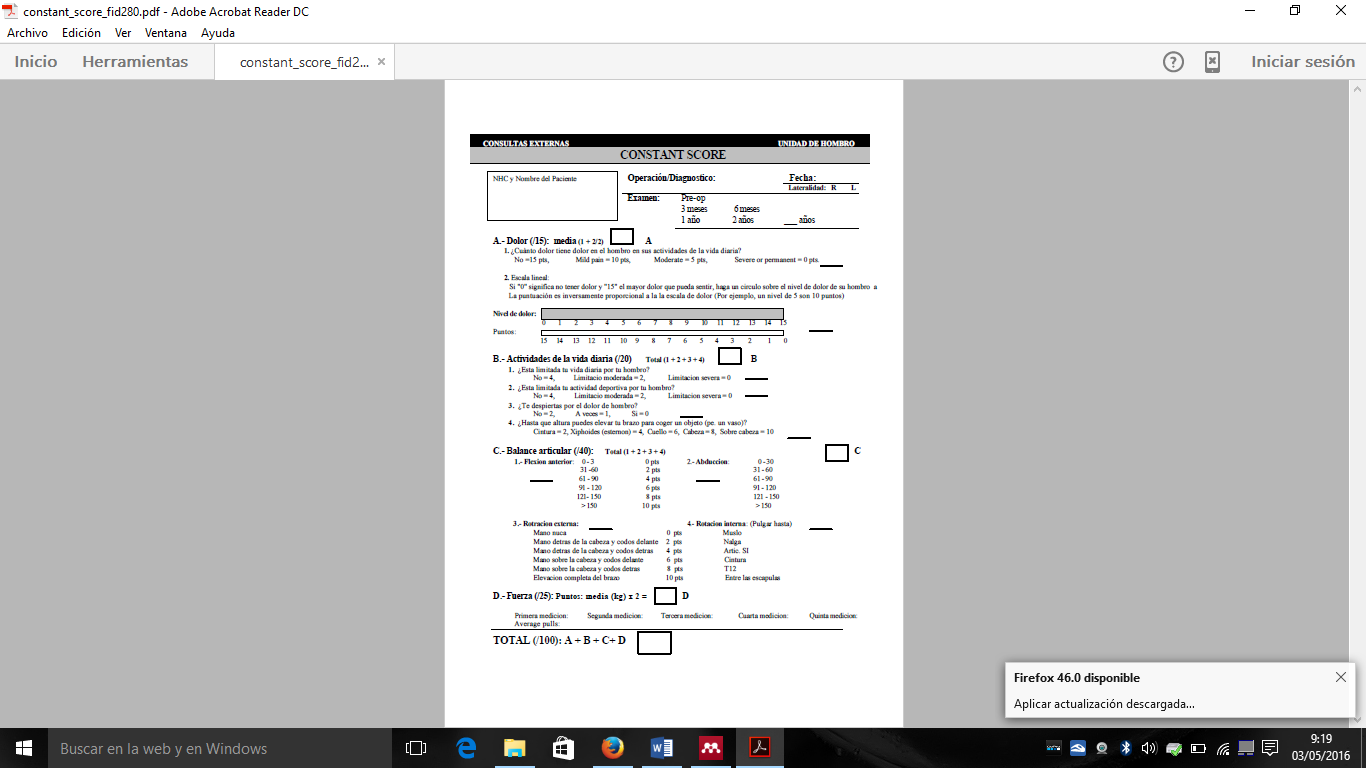


Additional File 1. Constant Murley Test Spanish Version

Supplement: Additional file 2: — Constant–Murley test, Spanish version. (DOCX 159 kb) [file 13063_2017_1822_MOESM2_ESM.docx]

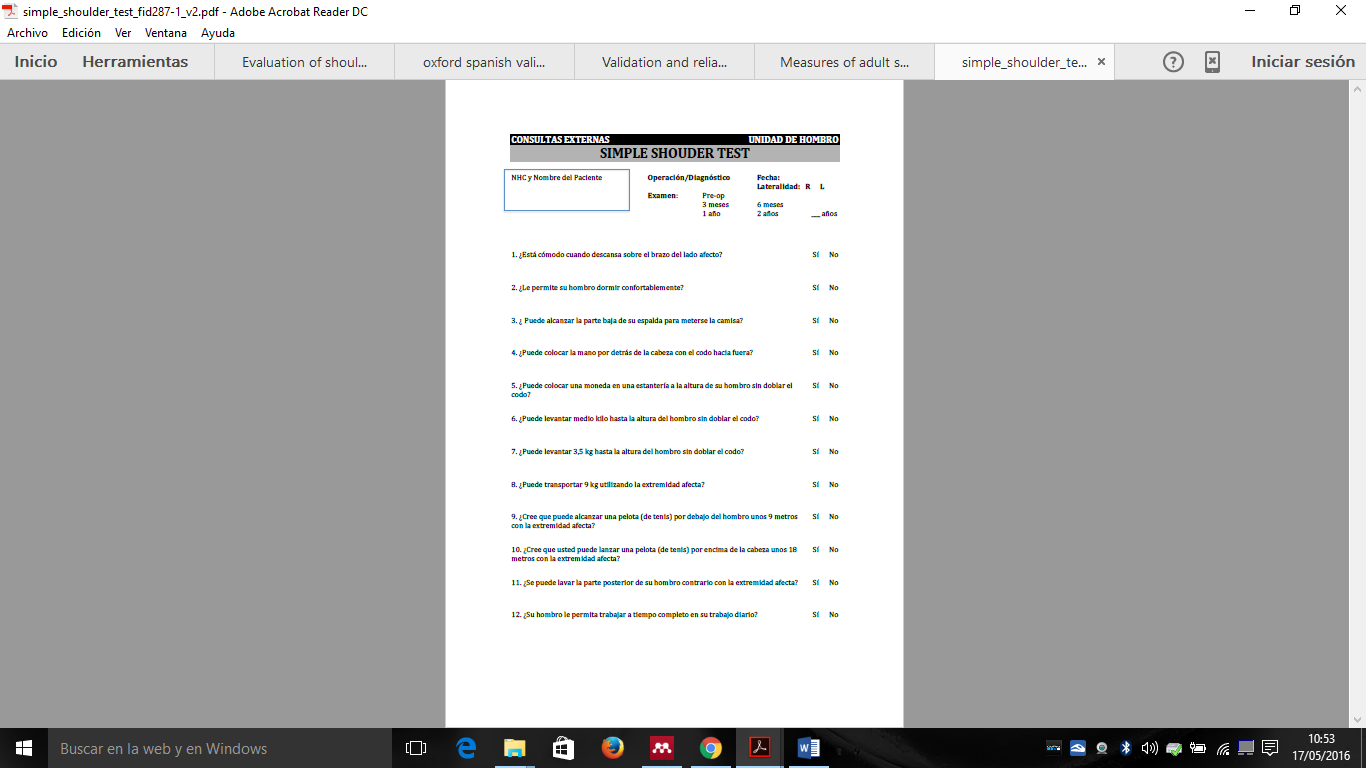


Additional File 2. Simple Shoulder Test Spanish Versión

Supplement: Additional file 3: — Simple Shoulder Test (SST), Spanish version. (DOCX 124 kb) [file 13063_2017_1822_MOESM3_ESM.docx]
