# Supplementary material for: Effectiveness of telerehabilitation programme following surgery in shoulder impingement syndrome (SIS): study protocol for a randomized controlled non-inferiority trial
Source: Trials. 2017 Feb 23;18:82. doi: 10.1186/s13063-017-1822-x (PMC5324280; doi:10.1186/s13063-017-1822-x)
Supplement: Additional file 4: — Telemedicine Satisfaction and Usefulness Questionnaire (TSUQ), Spanish version telerehabilitation adaption. (DOCX 35 kb) [file 13063_2017_1822_MOESM4_ESM.docx]

**Additional File 4. TUSQ Spanish Version**


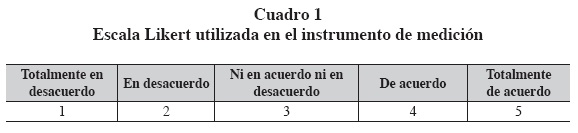
Se mide la percepción de los pacientes con la Telerehabilitación e incluye 17 preguntas que se valoran con una escala subjetiva de 5 puntos. El paciente obtiene puntuaciones de 17 a 85. A mayor puntuación, mejor percepción de la intervención de Telerehabilitación.

El Fisioterapeuta puede obtener una buena comprensión de mi problema de salud a través del ordenador.

La intervención de Telerehabilitación puede violar mi privacidad

El uso de los equipos necesarios para este programa de Telerehabilitación son difíciles de manejar para mí.

Puedo estar tan satisfechos al hablar con el fisioterapeuta a través del ordenador como al hablar en persona.

La Telerehabilitación puede mejorar mi salud en general.

La Telerehabilitación puede ahorrar tiempo para los Fisioterapeutas.

La Telerehabilitación no me puede ahorrar dinero.

Usando Telerehabilitación el Fisioterapeuta será capaz de controlar bien mi condición.

No me gusta que no hay contacto físico durante una visita de Telerehabilitación.

La Telerehabilitación es una forma conveniente de la prestación de atención médica para mí.

La intervención de Telerehabilitación me ahorra tiempo.

La Telerehabilitación será una forma estándar de prestación de asistencia sanitaria en el futuro.

La Telerehabilitación puede ser un añadido a la atención regular que recibo.

La Telerehabilitación puede reducir los costes para los sistemas de cuidado de la salud.

Un Fisioterapeuta no me puede examinar a través del ordenador como en persona.

La Telerehabilitación hace que sea más fácil para mí para comunicarme con el Fisioterapeuta.

No siempre puedo confiar en que el equipo funcione.
